# Development and Initial Feasibility of a Hospital-Based Acceptance and Commitment Therapy Intervention to Improve Retention in Care for Out-of-Care Persons with HIV: Lessons Learned from an Open Pilot Trial

**DOI:** 10.3390/jcm11102827

**Published:** 2022-05-17

**Authors:** Lilian Dindo, Ethan Moitra, McKenzie K. Roddy, Chelsea Ratcliff, Christine Markham, Thomas Giordano

**Affiliations:** 1 Department of Medicine, Baylor College of Medicine, Houston, TX 77030, USA; tpg@bcm.edu; 2 Houston VA HSR&D Center for Innovations in Quality, Effectiveness and Safety, Michael E. DeBakey Veterans Affairs Medical Center, Houston, TX 77030, USA; 3 Department of Psychiatry and Human Behavior, Brown University, Providence, RI 02906, USA; ethan_moitra@brown.edu; 4 Quality Scholars Program, VA Tennessee Valley Healthcare System, Nashville, TN 37212, USA; mckenzie.roddy@vumc.org; 5 Department of Psychology, Sam Houston State University, Huntsville, TX 77340, USA; chelsea.ratcliff@shsu.edu; 6 Department of Psychiatry & Behavioral Sciences, Baylor College of Medicine, Houston, TX 77030, USA; 7 Department of Health Promotion and Behavioral Sciences, University of Texas Health Science Center School of Public Health, Houston, TX 77030, USA; christine.markham@uth.tmc.edu

**Keywords:** hospital, HIV, Acceptance and Commitment Therapy, retention

## Abstract

Roughly 40% of persons with HIV (PWH) are not consistently involved in HIV care in the US. Finding out-of-care PWH is difficult, but hospitalization is common and presents an opportunity to re-engage PWH in outpatient care. The aims of this study were to (1) develop an Acceptance and Commitment Therapy (ACT)-based intervention for hospitalized, out-of-care PWH who endorse avoidance-coping to improve HIV treatment engagement; (2) examine the intervention’s initial feasibility and acceptability; and (3) to revise the study protocol (including the intervention), based on stakeholder feedback, in preparation for a randomized controlled trial (RCT) comparing ACT to treatment as usual. Therapists and HIV care experts developed a four-session ACT-based intervention to be delivered during hospitalization. Fifteen hospitalized patients with poorly controlled HIV enrolled in the open trial, eight completed four sessions, two completed three sessions, and seven provided qualitative feedback. Patients universally liked the intervention and the holistic approach to mental health and HIV care. Refinements included repeating key concepts, including representative graphics, and translating to Spanish. Among the patients who attended ≥3 ACT sessions, 5/10 attended a HIV-care follow-up visit and 5/7 who had labs had a viral load <20 2-months post-intervention. Next steps include conducting a randomized clinical trial exploring the impact of the refined intervention to treatment as usual on retention in care and viral load. ClinicalTrials.gov Identifier: NCT04481373.

## 1. Introduction

Human Immunodeficiency Virus (HIV) infection is a chronic condition that is treatable with Antiretroviral therapy (ART). Unfortunately, roughly 40% of people with HIV (PWH) in the United States are not consistently involved in HIV primary care [1,2,3]. For PWH, the U.S. Department of Health and Human Services (DHHS) recommends regular primary care medical visits (e.g., once every 3–6 months) after initiating care [4]. In fact, a widely used quality indicator requires at least two visits, separated by at least 90 days, within 12 months for established patients [5]. The recommended frequency for patients who recently initiated ART is higher still. Inadequate retention in HIV primary care affects access to ART and survival [6,7] and results in lower rates of HIV viral suppression [8]. Furthermore, many persons who are out of care have detectable viremia and are estimated to contribute to more HIV transmissions (43%) than the population in care but not suppressed (20%) and the undiagnosed population (38%) [9]. Poor retention in HIV care also exacerbates racial and ethnic disparities in health outcomes [10,11]. In summary, improving retention in care for PWH is of great public health significance.

Finding PWH who are out of care is challenging. A promising venue to locate and treat out-of-care PWH is the hospital setting. Hospitalizations are relatively common in PWH [12,13,14,15], with more than one in ten PWH being hospitalized each year [16,17]. Many out-of-care PWH are hospitalized with life-threatening yet preventable complications of inadequately treated HIV infections [13,18,19]. Indeed, hospitalization is more common among PWH who have CD4 cell counts < 200 c/uL and do not have viral suppression [16]. For example, in one large study of hospitalized PWH across four southern U.S. cities, the median CD4 count was 105 c/uL; only 65% were on ART, and only 14% had viral load suppression [18]. Moreover, the length of stay among this cohort can be long, with 69% of patients being hospitalized for ≥5 days [20]. Thus, hospitalization is a point of contact that an out-of-care PWH could have with the health care system outside the HIV clinic, providing an opportunity to enhance retention.

Many out-of-care PWH who are hospitalized do not successfully re-engage in care after discharge despite navigation services and other efforts to support continuity of care [21]. For instance, some hospitals might have embedded navigators to assist with re-linkage to care. Navigators can assist with overcoming structural barriers to care by assisting with appointment scheduling, linking patients to needed services after discharge, supporting applications for funding programs, and educating patients on the benefits of care. However, navigation does not appear to significantly improve outcomes. In our research [6] we found that at 6 months post-discharge, only 40% of persons were retained in care, only 51% had viral load improvement, and 8.4% died despite having navigation support from a hospital-based navigator as standard of care. In another study, hospitalized PWH who had been out of care for over a year received intensive service linkage support during their hospitalization. Nonetheless, only a third were linked to care within a month of discharge, and only 39% had viral suppression by 6 months [22]. These findings indicate that service linkage and navigation are insufficient, and different approaches are vital to improve retention in care for hospitalized PWH. To our knowledge, no efficacious interventions exist for out-of-care PWH who were recently hospitalized.

Our data from hospitalized PWH who are out of care suggest more attention is needed to address the psychosocial barriers that might undermine retention in care for recently hospitalized PWH, including HIV stigma, depressive feelings, and anxiety about health difficulties [23,24]. HIV-related self-stigma is incredibly common among PWH and is associated with a range of negative outcomes, including poor medication adherence, retention in medical care, and poor quality of life [25,26]. PWH also have significantly higher rates of co-occurring mental health diagnoses compared to the general population [27]. For example, over half of this population meets the criteria for major depressive disorder and/or generalized anxiety disorder [28,29], and these problems are even more prevalent in the out-of-care population of PWH [30].

**Figure 1:**
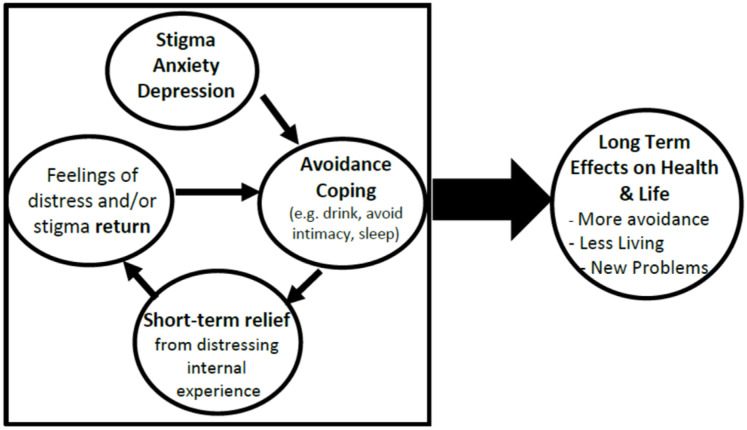
Long term effects of avoidance coping.

These psychosocial difficulties—stigma, depression, anxiety—are associated with avoidance-based coping, which can have negative long-term consequences [31]. For instance, if patients are avoidant due to fear of being stigmatized, they may avoid critical health behaviors, including attending clinic visits. They may also turn to harmful behaviors to cope, such as drug or alcohol use [32]. Although avoidant coping can lead to short-term relief, it can result in negative long-term consequences (See Figure 1). Evidence bears this out: avoidance coping is predictive of poor outcomes in PWH, including lower motivation to attend appointments and lower rates of viral suppression [3,4,5,6,7,8,9,10,11,12,13,14,15,16,17,18,19,20,21,22,23,24,25,26,27,28,29,30,31,32,33,34,35,36,37]. Addressing avoidance-based coping could improve HIV treatment outcomes [38].

**Figure 2:**
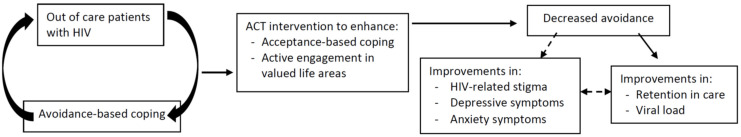
Conceptual model.

Acceptance and Commitment Therapy (ACT) is a transdiagnostic treatment that targets avoidance-based coping [39]. ACT helps patients overcome problematic avoidance, particularly avoidance of uncomfortable internal states and the situations that trigger such states. To counter avoidance of life’s challenges, patients are taught mindfulness, acceptance, and committed action towards valued life areas. Rather than address a specific symptom or disorder with a goal of symptom reduction, ACT helps people develop skills to engage more fully in valued life activities, even when there are painful emotions, troubling thoughts, and a strong motivation to escape or avoid them. As shown in Figure 2, we hypothesized that ACT would be well suited to decrease avoidance, which could lead to improved retention in care. Data show that ACT-based interventions can improve retention in care for newly diagnosed PWH [40,41] and improve ART adherence [36]. However, to our knowledge, ACT has not been used to improve retention among hospitalized and out-of-care PWH.

### The Present Study

Improving retention in care is essential for the survival of PWH as well as meeting the goal of “Ending the HIV Epidemic” (EHE; [42]) in the U.S. Hospitalization presents a unique opportunity in which clinicians might intervene to promote retention among out-of-care PWH. However, scant research has examined the hospital as a venue for intervention among PWH, with even less attention paid to interventions that might address their psychosocial barriers to retention in care. As such, our objectives in this open trial were to (1) develop an ACT-based intervention for hospitalized and out-of-care patients with HIV, (2) pilot the intervention with hospitalized PWH to determine the feasibility of recruiting out-of-care PWH in a hospital setting and acceptability of a brief ACT-based psychosocial intervention, (3) revise the study protocol (including the intervention), based on stakeholder feedback, in preparation for a randomized controlled trial (RCT) comparing ACT to treatment as usual. These objectives align with our long-term goal to develop easy-to-disseminate, low-intensity, brief interventions that can be administered in the context of hospital-based clinical services to improve the HIV care cascade.

## 2. Materials and Methods

### 2.1. Overview of Intervention

Four doctoral level psychologists (authors LD, EM, MR, CR), with expertise in ACT and clinical trials, developed a 4-session, ACT-based intervention called Targeting HIV Retention and Improved Viral load through Engagement (“THRIVE”). THRIVE was designed to be implemented individually and flexibly delivered to hospitalized PWH in hospital settings. That is, the therapist has the flexibility to conduct 1 session or more at a time, depending on each patient’s willingness and focus. We hypothesized that flexibility in session delivery would be needed to accommodate potentially unpredictable inpatient clinical situations and care services. The content of each session was intended to last 30–40 min. A therapist training manual and a patient manual were developed for this phase of the study. The patient manual was provided to patients at the beginning of the intervention and was used as a guide to the therapist–patient discussions. 

In summary, the aim of the intervention was to encourage patients to think about how caring for their HIV would positively impact other meaningful areas of their life. By cultivating an openness to difficult internal experiences and reducing avoidance, we aimed to increase healthy behaviors, particularly keeping healthcare appointments and taking HIV medications daily, and reduce the internal struggles associated with living with HIV. Throughout the intervention, patients were guided through several mindfulness practices as a way to practice present-moment awareness. The specific sessions included the following content.

Session 1 of the intervention focused on clarifying patient values and identifying internal obstacles that might be getting in the way of living a life consistent with those values, as well as working through a few exercises that explore the short- and long-term effects of avoidance-based coping. For example, patients were asked about their current status in the domains of Health, Love, Play, and Work and what they would like these areas of life to look like. Then, patients were asked about what gets in the way of doing what matters to them. After this, the therapist reviewed examples of how avoidance offers short-term relief (e.g., drinking alcohol to reduce anxiety) but results in greater distress and disability in the long term. At the end of session 1, patients were asked to complete two worksheets focused on values clarification prior to the next session. 

Session 2 was focused on providing education about HIV and best practices for managing HIV. Some PWH, especially those who are out-of-care, have little knowledge of their illness, how it may be effectively managed, and their own ability to influence its course. Thus, session 2 focused on educating patients on what HIV is, how it is (and is not) spread, what antiretroviral therapy (ART) is, how these medications are used, what the goals of ART are, how to remain healthy with HIV, and the impact of consistent health care on overall health. Session 2 ended with an exercise asking the patient to list two to three ways in which he/she would be better able to take care of his/her HIV. That is, patients were encouraged to identify actions that would improve their HIV status (e.g., keeping healthcare appointments, taking HIV medications daily, staying active) so that they can live a more meaningful life. The homework exercise provided after session 2 involved listing a value, goal, specific actions to achieve a goal, potential internal obstacles to achieving the goal (e.g., anxiety, stigma), and possible ways to better manage those internal obstacles. 

In Session 3, to challenge internalized HIV stigma, patients were taught to be more accepting and kinder to themselves. The session began with a brief self-compassion-focused mindfulness exercise and was followed with a debriefing and discussion of the meaning and importance of self-compassion. The remainder of the session focused on identifying specific actions that would align with values, brainstorming the barriers to these actions, highlighting the negative impact of avoiding health-based actions (e.g., patients were encouraged to examine the costs of stigmatization on their life, such as avoidance of medical care or intimate relationships), and discussing solutions to barriers. The homework involved writing out a goal and the barriers that may be anticipated. 

In session 4, patients were taught alternative skills to avoidance-based coping, including acceptance and cognitive defusion. For example, patients practiced “urge surfing” or acknowledging difficult emotions rather than struggling or avoiding them. Additionally, patients were taught that thoughts do not need to control actions and practiced cognitive defusion skills to help them gain emotional distance from thoughts. 

### 2.2. Recruitment Procedures

All subjects gave their informed consent for inclusion before they participated in the study. The study was conducted in accordance with the Declaration of Helsinki, and the protocol was approved by the Ethics Committee of the Baylor College of Medicine (Project identification code: H-47444, date: 5-13-2021). The open pilot enrolled individuals at Ben Taub Hospital, an acute care setting that is part of the Harris Health System in Houston, TX, USA. 

### 2.3. Inclusion/Exclusion Criteria

Because we were interested in real-world feasibility and delivering this intervention to individuals at highest risk for adverse HIV outcomes, eligible patients were (a) hospitalized at Ben Taub Hospital; (b) ≥18 years old; (c) able to speak English; (d) HIV infected; (e) able to provide informed consent and participate in the study; (f) those with an HIV viral load > 1000 c/mL; (g) never in care or currently out of HIV care, defined as not meeting the ‘visit constancy’ measure (≥1 completed HIV primary care visit in each of the three 4-month intervals preceding admission) or ≥2 “no shows” to HIV primary care visits in the last year; and (h) endorsing of one of the two avoidance coping statements with the highest factor loadings on the Avoidant Coping Subscale from the Coping with HIV/AIDS scale [43].

Exclusion criteria were (a) intending to use a source of HIV primary care other than our local HIV clinic after discharge; (b) in the opinion of the primary medical team caring for the patient, likely to be discharged to an institutional setting, die in the hospital, or enter hospice; (c) incarcerated or expected to be discharged to prison or jail; (d) enrolled in another research study with prospective follow-up; (e) pregnant, because pregnant women receive additional efforts to be linked and retained in care; and (f) admitted with acute psychosis, which would preclude informed consent or meaningful participation with the intervention.

### 2.4. Recruitment

**Figure 3:**
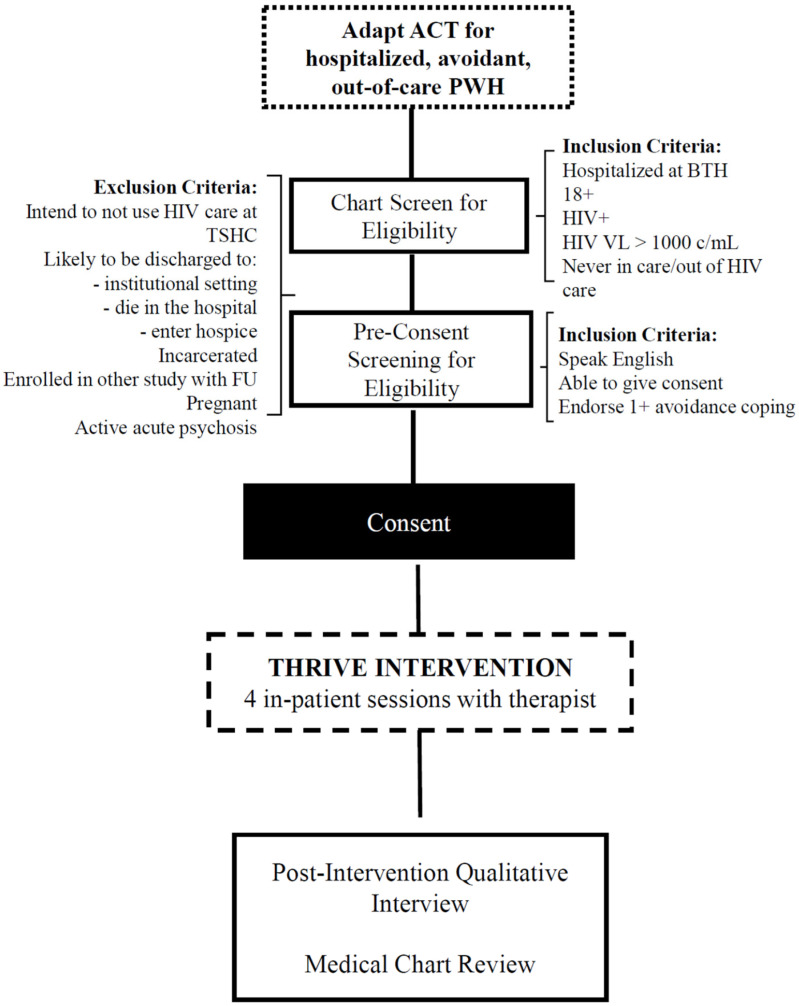
Summary of procedures.

With an IRB-approved waiver to review records, the research coordinator screened electronic health records using an automated filter that identified all patients currently hospitalized who had HIV listed as a diagnosis in their “problem list”. The coordinator then performed a brief chart review to preliminarily assess inclusion/exclusion criteria. If the patient appeared eligible, they were approached in the hospital to further determine eligibility and to discuss possible participation in the research project. If interested, they signed an informed consent form. Participants were compensated $20 for the baseline assessment and $20 for the post-intervention qualitative interview. Patients were not compensated for attending intervention sessions. See Figure 3 for a summary of the procedures. 

### 2.5. Measures

Feasibility. The feasibility of the study protocol was assessed using the following metrics: eligibility rate (percent of patients eligible at chart review screening that remain eligible after in-person screening and are offered informed consent by study staff), consent rate (percent of eligible patients offered informed consent who provided consent), enrollment rate (number of patients who consent per month), and session attendance rate (percent of participants who complete all four THRIVE sessions).

Acceptability. The acceptability of the study was assessed using qualitative interviews with patients (described below) as well as feedback from stakeholders (therapists, experts in HIV care, case managers, social workers, and researchers). 

Qualitative intervention feedback. Immediately following the intervention, a research coordinator conducted a brief structured interview (9 open-ended questions) to assess (a) barriers and facilitators to receiving the intervention in the hospital and utilizing learned skills, (b) how to improve the treatment protocol (e.g., strengths/weaknesses), (c) satisfaction with the intervention (e.g., most/least helpful), (d) usefulness of the patient manual, and (e) how the patient would describe the intervention to others. 

#### Self-Report Assessments Obtained at Baseline

Demographics and HIV data. We obtained demographic information from all participants, including age, marital status, gender identity, racial and ethnic background, employment, education, and student/work status. We also asked participants about their HIV history and lifetime history of risk behaviors associated with HIV infection (e.g., injection drug use).

Distress, coping, and stigma. *The Depression Anxiety and Stress Scale (DASS-21)* is a 21-item questionnaire that includes 3 subscales to assess current levels of depression, anxiety, and stress. It has demonstrated adequate construct validity and internal consistency [44]. *The Internalized AIDS-Related Stigma Scale (IARSS)* is a 6-item measure of internalized stigma, e.g., “I hide my HIV status from others” and “Being HIV positive makes me feel dirty”. Responses are “agree” or “disagree”, and the score is a count of the affirmative responses (range 0–6). The IARSS has been validated in U.S. and international populations [45,46]. *The Coping with HIV/AIDS Scale* is a 16-item scale that measures avoidance coping, positive coping, and seeking social support in patients with HIV [43]. The measure has been well validated and widely used, including internationally [47].

Electronic Health Record data. The medical charts of patients who enrolled in the study were reviewed to obtain information on active outpatient prescriptions for antiretroviral therapy, medical visits, and viral load (VL), following discharge from the hospital. 

### 2.6. Data Analysis

Descriptive statistics were used to summarize overall characteristics of the participants. All qualitative interviews were transcribed and reviewed by the 3 of the doctoral-level psychologists involved in the intervention development, 2 of whom conducted some interventions. Electronic medical records were reviewed to obtain lab results (VL) and outpatient care visits. 

## 3. Results

**Figure 4:**
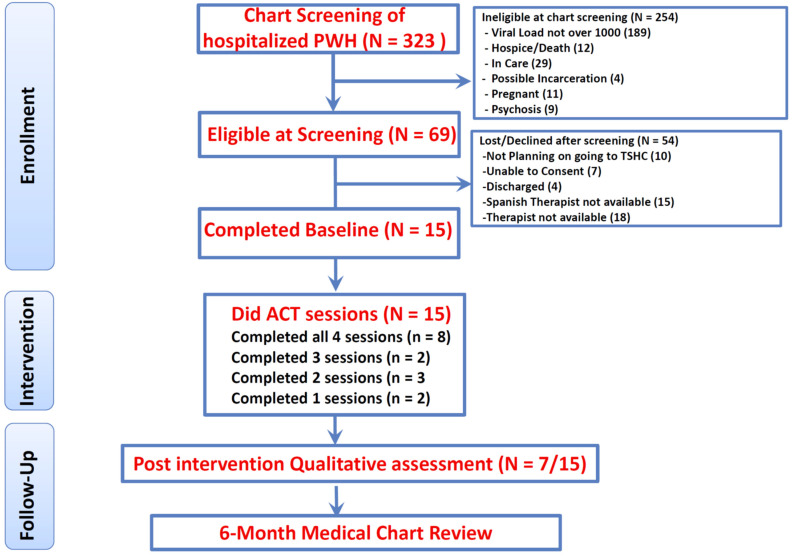
Consort diagram.

Between April 2021 and November 2021, there were 323 PWH hospitalized at the recruitment site. The electronic medical records of the 323 patients were reviewed, and 254 did not meet eligibility criteria, most commonly due to VL levels (78% had VL < 1000). These individuals were not approached, as their HIV was likely to be well managed. Sixty-nine hospitalized PWH were eligible at chart screening. These patients were approached and asked additional screening questions and informed about the current study. Approximately 50% could not be included in the study because they were Spanish speaking or because a therapist was not available due to the part-time staffing model for this preliminary study. Of the 69 eligible at screening, 15 PWH consented to complete the interventions (see Figure 4 for flow chart). No one who was otherwise eligible was excluded because they did not endorse avoidance coping. 

The 15 PWH who consented to the intervention. Fifteen patients with uncontrolled HIV were enrolled in this open trial. Patients were between the ages of 26 and 62. All 15 identified their sex as male at birth; one subsequently transitioned to female. Eleven of the 15 identified as heterosexual and four as gay. Most of the patients identified as Black, single, and disabled or unemployed. All the patients had an annual income of less than $25,000, with seven of them making less than $5000 annually. Only five of the patients had an education beyond high school. Of note, 5 of the 15 participants (33%) reporting living on the streets. On average, these patients were first told they were HIV positive 12 years ago (see Table 1). Two of the 15 patients reported a history of injection drug use. Four of the participants born male reported having sex with men but no injection drug use. One male patient reported a history of both injection drug use and sex with men. 

The participants were hospitalized for a variety of reasons, most of which could be related to poorly controlled HIV, including pneumonia (n = 3), skin and soft tissue infections (n = 3), systemic infections (n = 2), neurosyphilis, encephalitis, HIV-related nephropathy, penile ulcer, and parotid mass; other reasons for hospitalization were hyponatremia and pleural effusion. At the time of enrollment in the study, only 4 of the 15 participants had an active outpatient prescription for antiretroviral therapy, which is consistent with the study’s eligibility criteria that participants have a current VL > 1000 copies/mL and focus on persons who are not in HIV care. 

Mental Health, Coping, and Stigma. On the DASS-21 Depression subscale, seven patients reported symptoms in the normal range, one in the mild range, two in the moderate range, two in the severe range, and three in the extremely severe range (overall mean = 14.3, SD = 11.9). On the DASS-21 Anxiety subscale, six patients were in the normal range, five in the moderate range, and four in the extremely severe range (overall mean = 13.7, SD = 11.6). On the DASS-21 Stress subscale, nine patients were within the normal range, one was in mild range, one in the moderate, two in the severe, and two in the extremely severe range (overall mean = 16.4, SD = 11.5). Only four individuals scored within the normal range on all three scales. Thus, 73% of the patients had clinically elevated levels of depressive, anxiety, and/or stress symptoms. 

The avoidance coping scores were elevated and consistent with avoidance coping being used sometimes or a few times in the last month (mean = 16.8, SD = 3.9), similar to the scores found in our previous work with a similar population from Ben Taub Hospital [24]. The stigma scores ranged from 0 to the maximum 6, with a mean of 3.27 (SD = 2.02), which suggests the population is high in internalized stigma [45]. 

### 3.1. Acceptability and Feasibility

Table 2 provides a detailed summary of feasibility and acceptability benchmarks, barriers to each aspect of feasibility and acceptability that were observed in the present study, and how the study protocol was revised to address barriers. 

Two doctoral level therapists (authors LD and CR) with expertise in ACT and working with patients with chronic health problems, provided the therapy sessions in the hospital. Of the 15 patients enrolled in the study, eight (53%) completed all four intervention sessions, two completed three sessions, three completed two sessions, and two completed one session. There were seven instances when two sessions were completed during the same visit (e.g., session 1 and 2, or 3 and 4). Of the eight participants that completed all four sessions, seven also completed a follow-up qualitative interview. None of the other participants completed a qualitative interview. Of the seven participants who partially completed the intervention, five were discharged prior to intervention completion and were unreachable, one stated that it was too time consuming, and one stated that the compensation was not adequate. When one session was completed at a time, the mean length was 46 min (SD = 12.4). When two sessions were completed at a time, the mean length was 83 min (SD = 8.1). No adverse events occurred during the course of this open trial. The length of stay at the hospital ranged from 3 days to 21 days (Mean = 8.5, SD = 5.4). There was no apparent relation between living arrangements and completion of sessions. 

#### Follow-Up Outpatient Health Care Visits and VL

Twelve of the 15 participants were prescribed ART at the time of hospital discharge or shortly thereafter when they presented to the outpatient clinic. By 3 months follow-up, nine participants of the 15 remained active on ART. Of the 15 patients involved in the intervention, six attended a scheduled follow-up visit at Thomas Street Clinic within 2 months of discharge, and nine no-showed to their appointment or cancelled their appointment. Of note, among the six that attended a follow-up health care visits, three had completed all four sessions of the ACT intervention, two completed three sessions, and one completed one session (i.e., 5/10 patients who completed ≥3 ACT sessions attended a follow-up visit, and 1/5 patients who completed ≤3 ACT sessions attended a follow-up visit). 

Of the 15 patients involved in the intervention, eight obtained labs within 2 months of discharge. Of the eight who obtained labs, five had a VL < 20 (all five of whom had attended ≥3 ACT sessions), and three had a VL > 1000 (two of whom had attended ≥3 ACT sessions and one who attended 1 ACT session). In other words, 5/7 patients who attended ≥3 ACT sessions and obtained labs had a VL < 20 after the intervention. Conversely, 2/7 patients who attended ≥3 ACT sessions and obtained labs and the one patient who attended <3 ACT sessions and obtained labs had a VL > 1000 after the intervention. 

### 3.2. Analysis of Participant Feedback

As noted above, 7/8 patients who completed the intervention also provided qualitative feedback about the experience. Overall, patients indicated that they either did not know what to expect when they signed up for the study or that the intervention met/exceeded their expectations. They participated in the research study to learn more about what they were “dealing with” in terms of HIV, connect to resources that will help with treatments, help other patients like themselves, become motivated to care for their health, and talk to someone in a time of difficulty. These patients unanimously appreciated receiving the intervention in the hospital, stating that it was nice of the therapists to come to the patient, to have something to do in the hospital, and to receive one-on-one attention. Some patients stated that they wanted more sessions, and some stated that fewer would have been fine (see Table 3 for illustrative quotes).

When asked about their overall impressions about the sessions, patients indicated that they appreciated the holistic approach and the opportunity to share struggles beyond just HIV. 

Particular topics and skills that resonated with patients included deep breathing and grounding exercises, cognitive defusion or developing some distance from difficult thoughts, practicing self-compassion, acceptance of difficult emotions, clarifying values or what a meaningful life would look like, the importance of caring for their HIV, and how that may impact other areas of their life (see Table 3 for illustrative quotes).

When asked for suggestions to improve the intervention, patients did not provide much guidance. When asked if they would recommend the program to other patients, they unanimously indicated that they would. For example, one patient indicated he would describe the program in the following way:

“… the program is really, really beneficial. It helped me remember that I’m human, I make mistakes, and you know, the program is there to help you remember and know that the importance in life is health, and without taking your HIV medication I will end up in a situation where I’m in a hospital bed, and being in a situation where somebody’s helping me, trying to focus and remember the good things about healthy life, I mean it really helps a lot and it really helped me to really focus on my health and being out there in the world.”

(PT 7)

### 3.3. Revisions to the Study Protocol

A team of therapists, experts in HIV care, case managers, social workers, and researchers met to discuss the patient feedback, the experiences and feedback of therapists conducting the intervention, and recruiting challenges. Changes to the protocol were made based on these meetings and included (a) simplifying the intervention content (e.g., referencing one metaphor or experiential exercise that was well-received by patients in places that previously included several metaphors or exercises; adding session titles that highlight how each session relates to the other sessions); (b) repeating certain concepts to ensure retention of the information (e.g., each session underscored the idea that engaging in meaningful behaviors, even in the presence of difficult thoughts and feeling, allows patients to live a life they value); (c) translating the therapist and patient manuals into Spanish; (d) training a Spanish-speaking therapist in the intervention; (e) replacing many graphics to ensure more representation and sensitivity to the population; (f) revising some of the questions asked and language used to better reflect the circumstances of the patient population (e.g., inquiring about values in the domain of “interests” rather than “play”); and g) ensuring that therapists could be more flexible with their time at the hospital (see Table 2 for summary of revisions in the study protocol). 

The revised and simplified treatment manual is currently being used in the next (currently underway) phase of the project—A randomized clinical trial comparing the ACT intervention to Treatment as Usual. 

Session 1, titled “What Matters”, is focused on clarifying what people and things matter most to people and how HIV may impact these areas of life; identifying behaviors that reflect those valued areas; and discussing what thoughts and emotions get in the way of value-based behaviors. Session 2, titled “Doing What Matters”, is focused on HIV education and connecting how caring for their HIV can impact other domains of their life. Session 3, titled “Stuff that Gets in the Way”, reviews how thoughts and emotions get in the way of value-based behaviors and provides skills to deal with difficult thoughts and emotions, including self-compassion, mindfulness, acceptance, and defusion. Session 4, titled “Do it Anyway”, reviews acceptance and defusion techniques and connects these skills with the overall goal of engaging in life (i.e., engaging in meaningful behaviors even when it may be difficult in order to live a life the patient values). Each session also includes a grounding exercise. 

## 4. Discussion

To our knowledge, this is among the first acceptance and mindfulness-based retention interventions for out-of-care PWH specifically designed to be delivered in the hospital setting. Hospitals are an ideal venue to locate and treat PWH who are poorly retained in care, and PWH in the present study expressed a preference for meeting with therapists during hospitalization. The results demonstrate the acceptability of the intervention, as participants’ feedback was generally positive. In particular, PWH appreciated that the intervention provided a holistic approach to their health, including topics like mental health and wellbeing, in addition to HIV-specific content such as medication adherence. 

The results also suggest that the intervention is feasible, as therapists were able to deliver the intervention in the hospital setting. However, this open pilot trial highlighted several changes that need to be made to increase the feasibility of the intervention in future trials. Specifically, nearly a third of eligible patients could not participate because of Spanish language preference. As such, we developed a Spanish language version of our intervention to broaden our future reach. Additionally, a third of eligible patients could not participate because a therapist was not available during their hospital stay. The most common reason for consenting patients to not complete all intervention sessions was that patients were discharged and unreachable prior to completing the intervention. This speaks to how challenging it can be to engage with this underserved population and further highlights the importance of intervening rapidly and efficiently when they are hospitalized. To do this, it is critical to have a therapist available on site to ensure timely visits with the patients. The pilot nature of the current work did not allow full-time staffing, but subsequent work would need such a staffing model. Feasibility was also impacted by COVID-19, since institutional protocols did not allow face-to-face involvement of persons with COVID-19 into non-COVID-19 research, and the study team felt in-person interaction with the participants was critical. Nevertheless, this preliminary open trial provided a proof of concept that this intervention can be delivered in a hospital setting to out-of-care PWH. 

The present study also generated valuable information and experiences regarding revisions to the intervention to better suit the hospital setting and the population. The resulting revised intervention has streamlined content, more representative graphics, and examples and exercises tailored to the population. Additionally, examination of the open trial’s sample highlights the complex needs of out-of-care PWH. Within this small sample, a third were homeless, the majority were disabled and not working, and around three-quarters of the sample reported clinically significant depressive, anxiety, or stress symptoms. Incorporating an intervention like THRIVE, which takes a holistic approach to incorporating HIV care into care models that link patients with navigators and other resources may result in better outcomes for patients. 

Finally, the present study suggests that engagement in HIV outpatient care and viral load following hospital discharge are promising intervention outcomes to examine in future research. Though this trial was designed to examine feasibility and acceptability, data on post-discharge HIV appointment attendance and viral load were extracted from the electronic health records. These data suggest that patients who attended at least three of the four ACT sessions were more likely to attend a scheduled outpatient follow-up HIV care visit (5/10 patients) compared to patients who attended fewer than three ACT sessions (1/5 patients). Additionally, very limited data suggest that patients who attended at least three ACT sessions may be more likely to have a viral load of <20 (5/7, as only 7/10 completed labs) compared to patients who attended fewer than three ACT sessions (0/1, as only 1/5 completed labs). However, the present study was not designed to examine the intervention’s impact on retention in care or viral load, and therefore these very limited data should be considered as hypothesis-generating. 

## 5. Limitations

This was a small sample, which might not be representative of other out-of-care PWH. Due to the study design, participants who might seek care at our affiliated outpatient clinic were prioritized for recruitment, which may also limit generalizability. Additionally, as this study aimed to assess feasibility of recruitment and intervention delivery as well as patients’ acceptability of the intervention, self-reported follow up data were not obtained. Thus, though the present study indicates that a four-session ACT intervention delivered in a hospital setting is feasible and acceptable to patients, the intervention’s effect on patients’ mental health cannot be examined. Further testing is needed to examine this ACT intervention in a randomized controlled design now that it has been shown to be feasible and acceptable. 

## 6. Conclusions

These preliminary data on the feasibility and acceptability of THRIVE delivered in a hospital setting to out-of-care PWH support the continued investigation of this very brief ACT-based approach as an intervention to promote retention in care among PWH who are hospitalized and likely to be among the most ill. The next step is to deliver the revised intervention, along with the enhanced implementation procedures (e.g., greater therapist availability, Spanish speaking option), in a pilot randomized controlled trial to further demonstrate feasibility and acceptability and assess preliminary impact on retention in care (i.e., attendance in post-discharge outpatient appointments and viral load) as well as on patient-reported outcomes (e.g., depression, anxiety, and avoidance-based coping). The research team is currently working on such a randomized controlled trial, based on the findings and lessons learned from of the present study.

## Figures and Tables

**Table 1:** Description of the sample.

ACT (n = 15)	
Gender	
Male	11 (93%)
Female	1 (7%)
Age (Mean, SD)	48.7 (12)
Race/ethnicity	
African American	12 (80%)
Non-Hispanic White	2 (13%)
Hispanic	1 (7%)
Relationship Status	
Single	10 (67%)
Divorced	4 (27%)
Separated	1 (7%)
Education	
Did not complete high school	4 (27%)
GED/high school	6 (40%)
Some college or higher	5 (33%)
Employment	
Full or part time	2 (13%)
Unemployed	4 (27%)
Disabled	9 (60%)
Annual Income	
Less than $5000	7 (76%)
Between $5000 and $25,000	8 (24%)
Lving Arrangements	
Living in own home/apartment	4 (27%)
Living with family/friends	4 (27%)
Living on streets	5 (33%)
Homeless shelter	4 (7%)
Halfway home	1 (7%)
Years since HIV diagnosis (Mean, SD)	12 (9.9)
Length of hospital stay(days; Mean, SD)	8.5 (5.4)

**Table 2:** Feasibility and acceptability of study protocol.

Development Targets	Assessment Method	Benchmark	Open Pilot Result	Barriers Identified	Solution for Future RCT
Feasibility	Eligibility rate	50% of patients eligible at chart review screening (CRS) remain eligible after in-person screening and are offered informed consent by study staff	22% of patients eligible at CRS remained eligible after in-person screening and were offered informed consent by study staff	Most common reasons for patients eligible at CRS to be ineligible after in-person screening and not offered informed consent by study staff: 26% no therapist was available 22% needed Spanish-speaking therapist	Therapist needs to be embedded in the clinic to allow more flexible hours Intervention materials translated to Spanish; trained Spanish-speaking therapist to deliver intervention
Feasibility	Consent rate	50% of eligible patients offered informed consent provided consent	100% of eligible patients offered informed consent provided consent	No barriers identified	Current method of obtaining patients’ consent will be retained for RCT
Feasibility	Enrollment rate	2–3 patients per month	15 patients consented in 8 months (i.e., approximately 2 patients/month)	Barriers identified for recruitment rate (above) also served as barriers to enrolment rate	Given the high consent rate (100%), it is anticipated that the enrollment rate will increase to 8 patients per month in the RCT when barriers to recruitment are addressed (i.e., more flexible therapist hours; intervention offered in Spanish)
Feasibility	Session attendance rate	60% of consenting patients complete all 4 THRIVE sessions	53% of consented patients completed all 4 THRIVE sessions	33% of patients discharged before completing intervention and were unreachable	Intervention content simplified so that each session was shorter and easier to follow Therapists combine sessions when possibleTherapist embedded in hospital will allow more flexible scheduling
Acceptability	Qualitative interviews with participants	Majority of participants reported satisfaction with - In-hospital format - Session content - Session number and length	All participants noted that they appreciated in-hospital format and liked the session content Most (5/7) participants reported liking session number and length	Patients described few barriers in qualitative interviews, though many noted that it felt like a lot of information presented	Intervention content simplified and streamlined
Acceptability	Stakeholder feedback (i.e., patients, therapists, experts in HIV care, case managers, social workers, and researchers)	Majority of stakeholders indicate satisfaction with study protocol	Stakeholders noted several areas in which the intervention material could be revised to improve retention of content and acceptability	Therapist identified several places in which the intervention content could be simplified to help patients retain information Therapists identified several examples or word choices that did not seem directly relevant to patient population	Intervention content simplified and streamlined (e.g., only the most well-received metaphors/exercises retained, and core concepts repeated in each session) Intervention workbook graphics and wording revised to reflect experiences of the patient population

**Table 3:** Qualitative feedback about ACT intervention (n = 15).

Theme	Illustrative Quote(s)
Reasons for participation	“…I took the program to try to get myself information about what I’m up against, what I’m dealing with, and also to connect with resources that will help me find treatment for being HIV positive”. (PT 1)
	“Mainly trying to keep motivated to do more instead of just sitting back and letting things go by without pursuing what I can do instead”. (PT 5)
	“I wanted better ways to cope on taking my meds instead of being at the hospital bed… You know, I wasn’t on them—I mean if I was on them I wouldn’t be in the situation where I’m here in the hospital bed and struggling for my health right now”. (PT 7)
	“I needed like some therapist to talk to about it…that’s pretty much what it was therapist-wise…because I needed someone to share my pain, you know”. (PT 9)
	“I’ve never been a part of a research study so it was just more curiosity, just wondering what is gonna happen or what y’all were gonna talk about, what we’re gonna be doing”. (PT 10)
Did the intervention meet patients’ expectations?	“Oh, it’s wonderful. It’s much more than what I thought that it would be…. it’s very in depth, and it asks…. It asks personal questions, and then it helps you to work through”. (PT 1)
	“It was like right on point with where I needed to be right now”. (PT 9)
Thoughts on length of intervention and receiving it during hospitalization	“Well first of all, having spent a lot of time in the hospital you got nothing better to do..It’s so much easier whenever you’re in here because you just have extra—you have time on your hands..” (PT 10)
	“Maybe a little bit more sessions…in these four sessions I got a lot of information … or longer sessions, because like it’s a lot of good information, good pointers, and good conversations that we were having that helped me realize like the little things that could help with my anxiety, to help me remember to take my medicine, and knowing the fact that it’s okay that I’m dealing with stuff like this… even though I’m in a bed where I feel very negative, but it was helping me to help remember myself that I’m human, I make mistakes, I make problems, but it’s just looking past them and acknowledging them and finding out ways to like move on and realize what I did wrong, trying to avoid making the same mistake over and over again”. (PT 7)
Overall impression of the intervention	“It wasn’t all about HIV, you’re going to die, and all of that other crap that I thought that I was going to hear, and it was like oh, what type of person are you, and what do I see for myself…. The program was like don’t look like your life is over because you have HIV. It’s all in your head. It’s what you make of it”. (PT 7)
	“In a way I wanna say it was kind of embarrassing, but then it wasn’t embarrassing, it was just like I had a lot of things to talk about, you know, on my chest, especially dealing with HIV and not letting no one know that I have HIV for about 10 years, my social life and stuff like that, and like I can’t—you know, it’s kind of hard right now. So she helped me express that feeling or make sure I put it out there, and that she knows and things”. (PT 9)
The skills and knowledge that resonated with patients Breathe/be present Cognitive defusion Self-compassion Acceptance Values Importance of taking medication and going to medical appointments	“It was the exercise of the breathing… the anchor, because the anchor is basically like you… the breathing in and out, in and out, and just watching yourself just breathe actually helps refocus the mind and refocus on like what’s important or an easier way just to like not be overwhelmed and stressed with the tornado going around in your head with all these thoughts and just being able to like refocus, start from square one, and do it that way”. (PT 7)“I am having thoughts of feeling worthless. That doesn’t mean that I’m worthless. As opposed to saying that I am worthless. So you know. It gave an example of that, and then from there, it started opening up doors, and then it started giving me like breathing techniques. Ways of visualizing myself”. (PT 1)
	“Giving yourself more credit. That was one thing that stuck with me just because I never have, you know, it’s hard for me to… just like being easier on yourself. Letting yourself know, you know, you’re going through a hard time and that’s okay because everybody does”. (PT 10)
	“Well, what I learned from the sessions was we all struggle, we all are dealing with something, and we all can move past it…I’m scared of doing what I’m doing but I’m still gonna do it because I know it’s for the better of me and it helps me in the long run”. (PT 5)
	“A small life is somebody who won’t take their meds because they’re afraid of what other people are going to think. A big life is still having relationships, being honest with people about your HIV status, not feeling like that you don’t have to take your meds and not being afraid to take your meds”. (PT 1)
	“Just the importance of taking our medication for improving our health or way of life”. (PT 3)
	“The self-motivation, making sure I stay on top of my meds. You know, make sure I’m at the doctor’s appointments and things like that”. (PT 9)

## Data Availability

Data can be made available from the first author upon request.
